# Extended Arimoto–Blahut Algorithms for Bistatic Integrated Sensing and Communications Systems

**DOI:** 10.3390/e28010115

**Published:** 2026-01-18

**Authors:** Tian Jiao, Yanlin Geng, Zhiqiang Wei, Zai Yang

**Affiliations:** 1 School of Mathematics and Statistics, Xi’an Jiaotong University, Xi’an 710049, China; tianjiao@stu.xjtu.edu.cn (T.J.); zhiqiang.wei@xjtu.edu.cn (Z.W.); yangzai@xjtu.edu.cn (Z.Y.); 2 State Key Laboratory of ISN, Xidian University, Xi’an 710071, China

**Keywords:** integrated sensing and communication, capacity–distortion, Arimoto–Blahut algorithm

## Abstract

Integrated Sensing and Communication (ISAC) has emerged as a cornerstone technology for next-generation wireless networks, where accurate performance evaluation is essential. In such systems, the capacity–distortion function provides a fundamental measure of the trade-off between communication and sensing performance, making its computation a problem of significant interest. However, the associated optimization problem is often constrained by non-convexity, which poses considerable challenges for deriving effective solutions. In this paper, we propose extended Arimoto–Blahut (AB) algorithms to solve the non-convex optimization problem associated with the capacity–distortion trade-off in bistatic ISAC systems. Specifically, we introduce auxiliary variables to transform non-convex distortion constraints in the optimization problem into linear constraints, prove that the reformulated linearly constrained optimization problem maintains the same optimal solution as the original problem, and develop extended AB algorithms for both squared error distortion and logarithmic loss distortion. The numerical results validate the effectiveness of the proposed algorithms.

## 1. Introduction

Integrated sensing and communications (ISAC) has emerged as a key enabling technology for future wireless networks (beyond 5G and 6G), attracting extensive research across multiple domains. While much work has focused on wireless communication aspects such as waveform design and beamforming [1,2,3,4,5], a parallel line of information-theoretic research has recently developed to characterize its fundamental performance limits.

From an information-theoretic perspective, the performance of an ISAC system is quantified by its capacity–distortion function. This function is formulated as an optimization problem that maximizes mutual information under a distortion constraint. The authors in [6] examined the monostatic ISAC model and characterized the optimal trade-off between the capacity of reliable communication and the distortion of state estimation as an optimization problem with distortion constraints. A vector Gaussian channel with in-block memory was considered in [7,8], where the subspace trade-off and the random–deterministic trade-off between sensing and communication were identified. The capacity–distortion region of monostatic ISAC when the receiver has imperfect state knowledge was studied in [9]. The bistatic radar system, serving as a complementary paradigm to monostatic configurations, demonstrates superior channel interference mitigation capability due to its spatial diversity in transmitter–receiver separation. The authors in [10] characterized the rate-distortion and rate-detection exponent, respectively, of bistatic ISAC systems, where the sensing receiver estimates or detects the state based on the known sent information. Our previous work [11] considered a bistatic ISAC system in which the sensing receiver is unaware of the sent message. The capacity–distortion trade-off of the system was derived for some degraded channels and formulated as a rate-distortion optimization problem. In [12], a logarithmic loss (log-loss) function was selected to measure the quality of a soft estimate, and the corresponding capacity–distortion function of the bistatic ISAC model and the closed-form solutions for Gaussian channels under some conditions were derived. Therefore, solving the rate-distortion optimization problem is crucial for ISAC systems.

Unlike the tractable capacity–distortion trade-off in monostatic ISAC systems, which features a convex objective function and linear constraints, the corresponding problem in bistatic ISAC systems exhibits non-convexity in both its objective function and constraints. This inherent non-convex structure poses significant challenges to obtaining an efficient solution.

The Arimoto–Blahut (AB) algorithm, developed independently by Arimoto [13] and Blahut [14], is a widely applied method for calculating channel capacity and rate-distortion functions in information theory. To calculate the channel capacity of point-to-point channels, the AB algorithm replaces the conditional probability mass function (pmf) by a free variable and then maximizes the objective function over each variable alternatingly. The authors of [15] expanded the AB algorithm to compute the capacity region of the degraded broadcast channel, which is a non-convex optimization problem. This approach is also applicable for determining the capacity regions of less noisy broadcast channels. Furthermore, the authors of [16,17] developed AB-type algorithms to evaluate the supporting hyperplanes of the superposition coding region and those of the Nair–El Gamal outer bound for general broadcast channels. To calculate the rate-distortion function, Blahut [14] transformed the original distortion and compression rate problem into an unconstrained parameterized problem with respect to multipliers introduced by the distortion constraint. Then, for a fixed multiplier, a distortion and rate pair is calculated based on a framework similar to the AB algorithm above. Finally, the multiplier is traversed to obtain the complete rate-distortion function.

In contrast to the classical rate-distortion problem with its linear constraints, the capacity–distortion trade-off in bistatic ISAC systems is a non-convex optimization problem that creates difficulties for the application of the AB algorithm. This difficulty is compounded by the algorithm’s need to exhaustively search over Lagrangian multipliers, which complicates the direct determination of the achievable rate under a specific distortion constraint.

In this paper, we develop an optimization framework for rate-distortion optimization problems featuring non-convex yet differentiable distortion constraints, as motivated by emerging bistatic ISAC systems. Within this framework, we investigate the capacity–distortion trade-off under two typical distortion metrics: squared error (SE) and log-loss. The main contributions are summarized as follows:

- By introducing auxiliary variables, the original non-convex optimization problem is equivalently transformed into a convex-constrained form, with proven consistency in the optimal solutions. This provides a theoretical foundation for subsequent algorithm design.
- Based on the reformulated convex-constrained problem and the theory of the classical AB algorithm, an extended AB algorithm is proposed to obtain the optimal solution efficiently. This algorithm not only broadens the applicability of the AB approach but also supplies a feasible computational method for the capacity–distortion function in bistatic ISAC systems.
- The proposed algorithm directly determines the achievable rate under a given distortion constraint without exhaustively searching over Lagrangian multipliers, thereby addressing the computational inefficiency and practical limitations inherent in traditional AB-type algorithms for computing rate-distortion functions.
- Numerical results are provided to demonstrate the effectiveness of our proposed algorithm.

The rest of the paper is organized as follows. Section 2 introduces the monostatic and bistatic ISAC system models and capacity–distortion trade-off of ISAC systems. Section 3 proposes an extended AB algorithm for optimization problems of bistatic ISAC system with a convergence analysis. Section 4 generalizes the proposed algorithm to solve the lossy source coding problem with side information. Section 5 evaluates the performance of the proposed algorithms through numerical simulations. Section 6 concludes the paper.

Notations: Upper-case letters represent random variables, and lower-case letters represent their realizations. R and $R^{+}$ represent the sets of real numbers and non-negative real numbers, respectively. $X^{n}$ denotes the tuple of random variables $\left(X_{1},X_{2},\ldots ,X_{n}\right)$, E[X] denotes the expectation for a random variable *X*, and $X∼N\left(\mu ,\sigma^{2}\right)$ means that *X* is normally distributed, with mean μ and variance $\sigma^{2}$. H(·) denotes the entropy function, I(·;·) denotes the mutual information function, and D(·∥·) denotes the Kullback–Leibler divergence.

## 2. Problem Formulation

**Figure 1:**
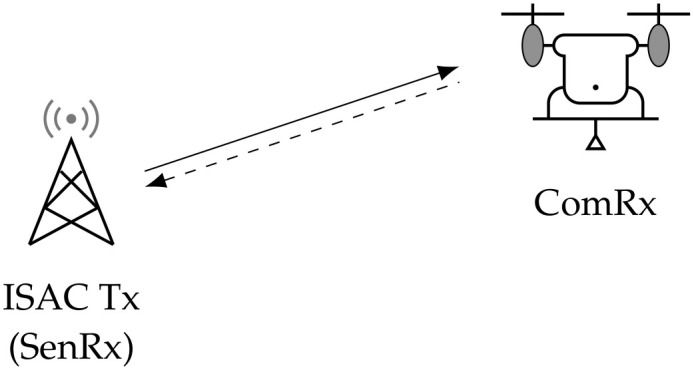
The monostatic ISAC system model.

**Figure 2:**
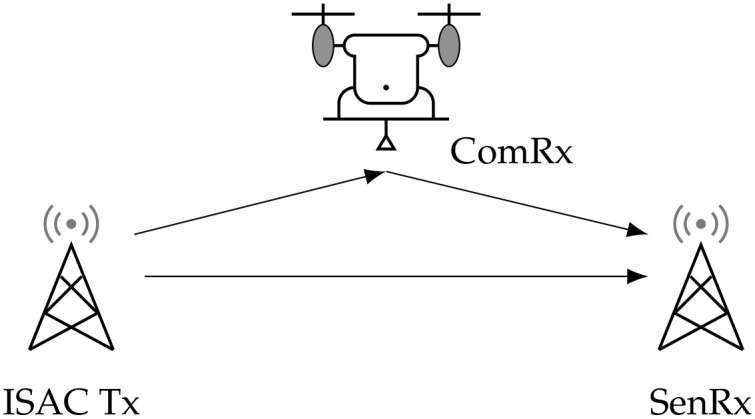
The bistatic ISAC system model.

In this section, we introduce the monostatic and bistatic ISAC systems and then present the capacity–distortion trade-off in the ISAC system with SE distortion and log-loss distortion, respectively. As shown in Figure 1, the monostatic ISAC system employs a dual-function transmitter (ISAC Tx) that simultaneously serves as the sensing receiver (SenRx), which is accompanied by a communication receiver (ComRx) [6]. In contrast, the bistatic ISAC system (Figure 2) adopts a distributed architecture with physically separated components: an ISAC Tx, a ComRx, and an independent SenRx. Specifically, the ISAC Tx transmits a codeword to convey information to the ComRx that knows the channel states perfectly. And then, the Tx also acts as a SenRx, estimating the channel state based on the received echo signals in the monostatic ISAC system. By contrast, in the bistatic ISAC system, the SenRx at another location receives both the radiated signals of the ISAC Tx and the reflected signals from the ComRx to perform the sensing task to estimate the channel states. Subsequently, we present the information-theoretic model for bistatic ISAC systems and formally define the capacity–distortion function.

**Figure 3:**
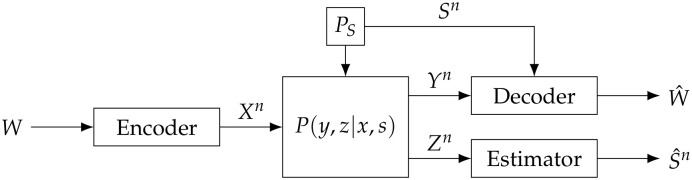
Two-receiver SDMC model.

As shown in Figure 3, the bistatic ISAC system is modeled as a state-dependent memoryless channel (SDMC) with two receivers [11], where the state sequence $S^{n}=\left(S_{1},\ldots ,S_{n}\right)$ is independent and identically distributed generated from a given state distribution $P_{S}\left(\cdot \right)$ and is assumed to be perfectly and noncausally available at the ComRx but unavailable at the SenRx. Specifically, the transmitter encodes the message *W* into a codeword $X^{n}$ and transmits it over the SDMC with two receivers. After receiving $Y^{n}$, the ComRx obtains the estimate of the message $\hat{W}$ by combining $Y^{n}$ with $S^{n}$. After receiving $Z^{n}$, the SenRx outputs ${\hat{S}}^{n}$ as the estimation of the state sequence $S^{n}$. The performance of the decoder is measured by the average probability of error $P_{e}^{\left(n\right)}=\mathrm{Pr}\left\{\hat{W}\neq W\right\}$. The accuracy of the state estimation is measured by the average expected distortion $D^{\left(n\right)}:=\frac{1}{n}E\left[d(S^{n},{\hat{S}}^{n})\right]=\frac{1}{n}\sum_{i=1}^{n}E\left[d(S_{i},{\hat{S}}_{i})\right]$, where $d:S\times \hat{S}\mapsto R^{+}$ is a bounded distortion function.

A pair (R,D) is said to be *achievable* if there exists a sequence of $\left(2^{nR},n\right)$ codes such that $\lim_{n\to \infty }P_{e}^{\left(n\right)}=0,{\bar{\lim}}_{n\to \infty }D^{\left(n\right)}\leq D$. The capacity–distortion function is defined as C(D)=max{R:RisachievableforthegivenD}, and this definition also applies to the monostatic ISAC system. To avoid confusion, we denote the capacity–distortion function of the monostatic ISAC system as $C_{m}\left(D\right)$. Furthermore, the capacity–distortion function of the ISAC system has various forms for different distortion metrics. In the following, we take SE (SE focuses on the magnitude of prediction error and is well suited for point estimation and quadratic cost problems) and log-loss (log-loss captures the probability consistency between the true and estimated distributions, making it suitable for distribution estimation and likelihood-based inference) distortion as examples to show the specific capacity–distortion function for the monostatic and bistatic ISAC systems.

### 2.1. ISAC System with SE Distortion

When the distortion metric is selected as SE, i.e., $d\left(s,\hat{s}\right)={\left(s-\hat{s}\right)}^{2}$, we have the following capacity–distortion function results for both the monostatic and bistatic ISAC systems.

**Lemma** **1** (Theorem 1, [6]). *The capacity–distortion function $C_{m}\left(D\right)$ for the monostatic ISAC system is the optimal solution to the following optimization problem:*

(1) $$\begin{array}{cc} \max_{P_{X}} & \;I(X;Y|S)\\ s.t. & \;E\left[{\left(S-\hat{S}\left(X,Z\right)\right)}^{2}\right]\leq D, \end{array}$$

*where $\hat{S}\left(x,z\right)=\arg\min_{S^{\prime }}\sum_{s}P_{S|XZ}\left(s|x,z\right){\left(s-s^{\prime }\right)}^{2}$, and the joint distribution of SXYZ is given by $P_{X}P_{YZS|X}$ for some pmf $P_{X}$.*

As demonstrated, the constraint term in problem (1) is linear with respect to the optimization variable $P_{X}$, since

$$P_{S|XZ}\left(s|x,z\right)=\frac{P_{X}\left(x\right)P_{ZS|X}\left(z,s|x\right)}{\sum_{s}P_{X}\left(x\right)P_{ZS|X}\left(z,s|x\right)}=\frac{P_{ZS|X}\left(z,s|x\right)}{\sum_{s}P_{ZS|X}\left(z,s|x\right)},$$

which is independent of $P_{X}$. Thus, the constraint term in problem (1) can be rewritten as $\sum_{x}P_{X}\left(x\right)c\left(x\right)\leq D$, where $c\left(x\right)=\sum_{z,s}P_{ZS|X}\left(z,s|x\right){\left(s-\hat{s}\right)}^{2}$ is independent of $P_{X}$.

**Lemma** **2** (Theorem 6, [11]). *For the bistatic ISAC system, the capacity–distortion function C(D) for the case where X−(Y,S)−Z forms a Markov chain is the optimal solution to the following optimization problem:*

(2) $$\begin{array}{cc} \max_{P_{UX}} & \;I(X;Y|U,S)+I(U;Z)\\ s.t. & \;E\left[{\left(S-\hat{S}\left(U,Z\right)\right)}^{2}\right]\leq D, \end{array}$$

*where $\hat{S}\left(u,z\right)=\arg\min_{S^{\prime }}\sum_{s}P_{S|UZ}\left(s|u,z\right){\left(s-s^{\prime }\right)}^{2}$, and the joint distribution of SUXYZ is given by $P_{UX}P_{YZS|X}$ for some pmf $P_{UX}$.*

It is observed that the objective function in the optimization problem (2) is the sum of two mutual information terms, which is non-convex with respect to the optimization variable $P_{UX}$. In addition, the constraint term in problem (2) is also non-convex with respect to the optimization variable $P_{UX}$ by observing that

$$E\left[{\left(S-\hat{S}\left(U,Z\right)\right)}^{2}\right]=\sum_{s,u,z,x}P_{UX}\left(u,x\right)P_{ZS|X}\left(z,s|x\right){\left(s-\hat{s}\left(u,z\right)\right)}^{2}$$

and

$$P_{S|UZ}\left(s|u,z\right)=\frac{\sum_{s}\sum_{x}P_{UX}\left(u,x\right)P_{ZS|X}\left(z,s|x\right)s}{\sum_{x}P_{UX}\left(u,x\right)P_{Z|X}\left(z|x\right)},$$

which makes the problem (2) difficult to solve via existing methods.

### 2.2. ISAC System with Log-Loss Distortion

If the distortion metric is log-loss, i.e., $d\left(s,\hat{s}\right)=-\log\hat{P}\left(s\right)$, where $\hat{S}=\hat{P}\left(S\right)$ is the soft estimator of *S*, the following results hold.

**Lemma** **3** (Theorem 1, [18]). *The capacity–distortion function $C_{m}\left(D\right)$ for the monostatic ISAC system is the optimal solution to the following optimization problem:*

(3) $$\begin{array}{cc} \max_{P_{X}} & \;I(X;Y|S)\\ s.t. & \;H(S|X,Z)\leq D, \end{array}$$

*where the joint distribution of SXYZ is given by $P_{X}P_{YZS|X}$ for some pmf $P_{X}$.*

Note that the constraint term in problem (3) is also linear with respect to the optimization variable $P_{X}$, since the term $P_{S|XZ}$ is independent of $P_{X}$.

**Lemma** **4** (Corollary 2, [12]). *For the bistatic ISAC system, the capacity–distortion function C(D) for the case where X−Y−Z forms a Markov chain is the optimal solution to the following optimization problem:*

(4) $$\begin{array}{cc} \max_{P_{UX}} & \;I(X;Y|U)+I(U;Z)\\ s.t. & \;H(S|U,Z)\leq D, \end{array}$$

*where the joint distribution of SUXYZ is given by $P_{UX}P_{Y|X}P_{ZS|X}$ for some pmf $P_{UX}$.*

It is observed that the constraint term in the optimization problem (4) remains non-convex with respect to the optimization variable $P_{UX}$, which poses a challenge to the problem solution. In addition, note that the Markov chain in Lemma 4 differs from the Markov chain in Lemma 2, which stems from the different settings for the sensing parameters in the bistatic ISAC model in [12] and Figure 2. One of the sensing targets is the ComRx, while the other is a target independent of the ComRx. Nevertheless, there is no fundamental difference between their information theory models. Therefore, to facilitate subsequent comparison with the theoretical results in [12], we adopt the model and theorem results proposed in [12] when the distortion metric is log-loss.

## 3. Extended AB Algorithm for Bistatic ISAC

Before introducing the extended AB algorithm, we review the classical AB algorithm, which was originally developed to compute the channel capacity of discrete memoryless point-to-point channels. For a discrete memoryless point-to-point channel with input *X* and output *Y*, its channel capacity is given by

(5) $$\begin{array}{c} C=\max_{p(x)}I\left(X;Y\right), \end{array}$$

where p(x) is the input distribution. Based on the mutual information formula

$$\begin{array}{c} I\left(X;Y\right)=\sum_{x,y}p\left(x\right)p\left(y|x\right)\ln\frac{p(x|y)}{p(x)}:=F\left(p\right), \end{array}$$

consider the expression

$$\begin{array}{c} \sum_{x,y}p\left(x\right)p\left(y|x\right)\ln\frac{q(x|y)}{p(x)}dxdy=\sum_{x}p\left(x\right)\left(\sum_{y}p\left(y|x\right)\ln q\left(x|y\right)-\ln p\left(x\right)\right):=F\left(p,q\right), \end{array}$$

where p(x|y) is replaced by a new variable q(x|y). The following result serves as a starting point of the classical AB algorithm.

**Lemma** **5** (Theorem 1, [14]). *The following properties hold:*

*(a)*  *$C=\max_{p(x)}\max_{q(x|y)}F\left(p,q\right)$.*
*(b)*  *For fixed p(x), the function q↦F(p,q) is maximized by q(x|y)=p(x|y).*
*(c)*  *For fixed q(x|y), the function p↦F(p,q) is maximized by*

$$\begin{array}{c} p\left(x\right)=\frac{e^{\sum_{y}p\left(y|x\right)\ln q\left(x|y\right)}}{\sum_{x}e^{\sum_{y}p\left(y|x\right)\ln q\left(x|y\right)}}. \end{array}$$

Based on the above result, the mutual information maximization problem (5) can be transformed into two maximization subproblems, where each subproblem admits a closed-form update. This leads to the development of the AB algorithm, which performs an alternating update of the two subproblems. It is worth noting that the AB algorithm is designed for discrete distributions. For continuous distributions, it is usually necessary to convert them into discrete distributions through quantization before applying the AB algorithm.

In the following, we focus on solving the optimization problems (2) and (4). Recalling the optimization problem for the monostatic ISAC system in Lemmas 1 and 4, where the constraint terms are linear in the optimization variable and the objective function is concave with respect to the optimization variable, the AB algorithm can be applied to derive the result in [6] (Theorem 4), where for each fixed multiplier μ introduced by the distortion constraint, a rate and distortion pair is derived, and the capacity–distortion function is obtained by traversing the multiplier. However, for the bistatic ISAC system, as previously discussed, the non-convexity of both the constraint terms and the objective function prevents the direct application of the AB algorithm to obtain an analogous algorithm. Fortunately, by introducing a special free variable (c(u,z) in (10) or f(u,s,z) in (16)), we can transform this complex optimization problem into a bivariate optimization problem, in which one subproblem is a linearly constrained mutual information maximization problem, which can then be solved using the AB algorithm.

### 3.1. Algorithm for Optimization Problem *(2)*

In this subsection, we focus on solving the optimization problem (2). For convenience, we omit the subscripts in the probability distribution and replace $P_{UX}\left(u,x\right)$ with p(u,x) in the following derivation. In addition, all summation symbols without an index represent the sum of all variables in the expression.

Note that the non-convex constraint in the optimization problem constitutes the key obstacle to solving this problem using the existing AB algorithm. To address this issue, we introduce a new variable c(u,z) that transform the non-convex constraint into a linear constraint, and the corresponding optimization problem with the linear constraint is as follows:

(6) maxp(u,x)c(u,z)∑p(u,x)p(y,s,z|x)logp(x|u,y,s)p(u|z)p(u,x)s.t.∑p(u,x)p(z,s|x)(s−c(u,z))2≤D,∑p(u,x)=1.

For the optimization problem (6), we have the following result.

**Theorem** **1.** *The optimal solution of the original optimization problem *(2) *is the same as that of the optimization problem *(6)*.*

**Proof of Theorem** **1.** The objective function in the original optimization problem (2) is as follows:

$$\begin{array}{cc} \; & F\left(p(u,x)\right)=I\left(X;Y|U,S\right)+I\left(U;Z\right)\\ = & \sum p\left(u,x\right)p\left(y,s,z|x\right)\log\frac{p(x|u,y,s)p(u|z)}{p(u,x)}. \end{array}$$

By comparing the original optimization problem (2) with the new optimization problem (6), we observe that the feasible set of the problem (2) is a subset of the feasible set of the problem (6). Therefore, the maximum value of the objective function in the optimization problem (2) is less than or equal to that in the problem (6). On the other hand, the Lagrangian function corresponding to the optimization problem (6) is

$$\begin{array}{cc} L(p(u,x),c(u,z),\lambda ,\alpha ) & =F\left(p(u,x)\right)+\lambda \left(D-\sum p\left(u,x\right)p\left(z,s|x\right){\left(s-c\left(u,z\right)\right)}^{2}\right)\\ & \;+\alpha \left(\sum p(u,x)-1\right), \end{array}$$

where λ and α are multipliers introduced for the constraints. Denote the optimal solution of the optimization problem (6) by $\left(p^{*},c^{*}\right)$. According to the Karush–Kuhn–Tucker (KKT) condition, we have $L_{c(u,z)}^{\prime }\left(p^{*},c^{*}\right)=0$. Thus, we get

$$\begin{array}{c} \sum_{x,s}p^{*}\left(u,x\right)p\left(z,s|x\right)c^{*}\left(u,z\right)=\sum_{x,s}p^{*}\left(u,x\right)p\left(z,s|x\right)s, \end{array}$$

i.e., $c^{*}\left(u,z\right)=\sum_{s}p\left(s|u,z\right)s$. Given that the estimator satisfies $\hat{s}\left(u,z\right)=\arg\min_{S^{\prime }}\sum_{s}P_{S|UZ}$$\left(s|u,z\right){\left(s-s^{\prime }\right)}^{2}=\sum_{s}P\left(s|u,z\right)s=c^{*}\left(u,z\right)$, it follows that $\left(p^{*},c^{*}\right)$ is also a feasible solution to the original optimization problem (2), thereby completing the proof.    □

In the following, we proceed to solve optimization problem (6). Building upon the derivation of Theorem 1, we obtain the optimal form of the variable c(u,z). For fixed c(u,z), the problem reduces to a mutual information maximization with linear constraints, which can be reformulated as a bilevel optimization problem by applying the framework of the AB algorithm. Specifically, we define

(7) $$\begin{array}{c} \tilde{F}\left(p(u,x),q(x|u,y,s),q(u|z)\right)=\sum p\left(u,x\right)p\left(y,s,z|x\right)\log\frac{q(x|u,y,s)q(u|z)}{p(u,x)}, \end{array}$$

which is a concave function of the optimization variable p(u,x) for fixed q(x|u,y,s) and q(u|z). Furthermore, we obtain that

$$\begin{array}{cc} \; & F\left(p(u,x)\right)-\tilde{F}\left(p(u,x),q(x|u,y,s),q(u|z)\right)\\ = & \sum p\left(u,x\right)p\left(y,s|x\right)D\left(p(x|u,y,s)∥\;q(x|u,y,s)\right)+\sum p\left(u,z\right)D\left(p(u|z)∥\;q(u|z)\right)\geq 0, \end{array}$$

where the equality holds when q(x|u,y,s)=p(x|u,y,s) and q(u|z)=p(u|z). This means that $F\left(p(u,x)\right)=\max_{q(x|u,y,s),q(u|z)}\tilde{F}\left(p(u,x),q(x|u,y,s),q(u|z)\right)$. Therefore, the original optimization problem (2) is equivalent to the following optimization problem:

(8) maxp(u,x)c(u,z)maxq(·|·)∑p(u,x)p(y,s,z|x)logq(x|u,y,s)q(u|z)p(u,x)s.t.∑p(u,x)p(z,s|x)(s−c(u,z))2≤D,∑p(u,x)=1,

where the optimal q(·|·) satisfies

(9) $$\begin{array}{c} q(x|u,y,s)=p(x|u,y,s)\;\mathrm{and}\;q(u|z)=p(u|z), \end{array}$$

and the optimal c(u,z) satisfies

(10) $$\begin{array}{c} c\left(u,z\right)=\hat{s}\left(u,z\right)=\frac{\sum_{s,x}p\left(u,x\right)p\left(z,s|x\right)s}{\sum_{x}p\left(u,x\right)p\left(z|x\right)}. \end{array}$$

It is observed from (10) that the optimal value of auxiliary variable c(u,z) corresponds to the minimum mean SE (MMSE) estimator of the given distribution p(u,x). Then, we derive the optimal variable *p* of problem (8). The Lagrangian function corresponding to the optimization problem (8) is

(11) $$\begin{array}{cc} \; & L(p(u,x),c(u,z),q,\lambda ,\alpha )\\ = & \tilde{F}\left(p(u,x),q(x|u,y,s),q(u|z)\right)+\lambda \left(D-\sum p\left(u,x\right)p\left(z,s|x\right){\left(s-c\left(u,z\right)\right)}^{2}\right)\\ & \;+\alpha \left(\sum p(u,x)-1\right), \end{array}$$

where λ and α are multipliers introduced for the constraints. By setting the gradient of (11) with respect to the optimization variable p(u,x) to zero, we get

(12) $$\begin{array}{c} p\left(u,x\right)=\frac{e^{d[q](u,x)-\lambda w(u,x)}}{\sum_{u,x}e^{d[q](u,x)-\lambda w(u,x)}}, \end{array}$$

where

$$\begin{array}{c} d\left[q\right]\left(u,x\right)=\sum_{y,s}p\left(y,s|x\right)\ln q\left(x|u,y,s\right)+\sum_{z}p\left(z|x\right)\ln q\left(u|z\right) \end{array}$$

and $w\left(u,x\right)=\sum_{s,z}p\left(z,s|x\right){\left(s-c(u,z)\right)}^{2}$. Furthermore, since the capacity–distortion function of the system is monotonically non-decreasing with respect to the distortion *D* [11] (Lemma 1), the optimal solution of the optimization problem must be found on the boundary of the distortion constraint set. In other words, the distortion constraint is satisfied in an equality form at the optimal solution. Therefore, λ satisfies

(13) $$\begin{array}{c} G\left(\lambda \right):=D-\sum_{u,x}\frac{e^{d[q](u,x)-\lambda w(u,x)}}{\sum_{u,x}e^{d[q](u,x)-\lambda w(u,x)}}w\left(u,x\right)=0. \end{array}$$

Let $g_{\lambda }\left(u,x\right)=d\left[q\right]\left(u,x\right)-\lambda w\left(u,x\right)$; then, we get

$$\begin{array}{cc} \; & G^{\prime }\left(\lambda \right)=-\frac{\sum e^{g_{\lambda }\left(u,x\right)}\left(-w^{2}\left(u,x\right)\right)\sum e^{g_{\lambda }\left(u,x\right)}}{{\left(\sum e^{g_{\lambda }\left(u,x\right)}\right)}^{2}}+\frac{\sum e^{g_{\lambda }\left(u,x\right)}w\left(u,x\right)\sum e^{g_{\lambda }\left(u,x\right)}\left(-w(u,x)\right)}{{\left(\sum e^{g_{\lambda }\left(u,x\right)}\right)}^{2}}\\ \; & =\frac{\sum e^{g_{\lambda }\left(u,x\right)}w^{2}\left(u,x\right)\sum e^{g_{\lambda }\left(u,x\right)}-{\left(\sum e^{g_{\lambda }\left(u,x\right)}w\left(u,x\right)\right)}^{2}}{{\left(\sum e^{g_{\lambda }\left(u,x\right)}\right)}^{2}}\geq 0, \end{array}$$

where the last inequality holds according to the Cauchy–Schwarz inequality. Thus, the equation G(λ)=0,λ≥0 has a unique solution when *D* is achievable. Furthermore, based on the analysis in [11], we obtain that the minimum achievable distortion is $D_{\min}=\min_{p(x)}\mathrm{Var}\left(S|X,Z\right)$. This corresponds to the case where the sent information is fully decoded at the SenRx to assist in estimation, yielding a minimum distortion identical to that of a monostatic ISAC system. The maximum achievable distortion is $D_{\max}=\mathrm{Var}\left(S|Z\right)$, where the input distribution p(x) is selected to maximize the communication rate I(X;Y|S). In this case, the SenRx does not perform decoding and instead uses the received signal directly to estimate the state, thereby maximizing the estimation error while achieving the same maximum communication rate as a monostatic ISAC system. Therefore, we obtain that the achievable distortion range is $\left[D_{\min},D_{\max}\right]$. Based on the derivation above, we present the proposed extended AB algorithm for optimization problem (2) in Algorithm 1, where the original variable *p* and the additionally introduced variables *q* and *c* are updated in closed form.
**Algorithm 1** Extended AB algorithm for the optimization problem (2)**Input:** 
p(y|x,s), p(z|x,s), p(s).  1:Compute $D_{\min}$ and $D_{\max}$ and select $D\in [D_{\min},D_{\max}]$.  2:Initialize p(u,x),c(u,z).  3:**while** not converge **do**  4:   Update q(x|u,y,s) and q(u|z) based on (9).  5:   Solve λ based on the equation (13).  6:   Update p(u,x) based on (12).  7:   Update c(u,z) based on (10).  8:**end while****Output:** 
p(u,x).

It is worth noting that the newly introduced variable c(u,z) in our optimization problem effectively replaces the estimator $\hat{s}\left(u,z\right)$ in the constraint—involving a similar operation that is also performed for the case of log-loss distortion—as demonstrated later. This substitution is motivated by two key reasons: Non-convexity elimination: The non-convexity of the constraints caused by the non-convexity of the estimator with respect to the optimization variable is mitigated by relaxing the estimator into a free variable, thereby transforming the non-convex constraints into linear ones. ‌Optimality preservation‌: The optimality of the estimator ensures that the update expression for this free variable exactly matches the form of the original estimator, as shown in (10), guaranteeing both solution quality and algorithm convergence.

**Remark** **1.** *For the Gaussian channel model with a power constraint for the bistatic ISAC system, the corresponding optimization problem with an SE distortion constraint and a power constraint is*

(14) $$\begin{array}{cc} \max_{P_{UX}} & \;I(X;Y|U,S)+I(U;Z)\\ s.t. & \;E\left[{\left(S-\hat{S}\left(U,Z\right)\right)}^{2}\right]\leq D,\\ & \;E[X^{2}]\leq B. \end{array}$$

*For this optimization problem, Algorithm 1 can be applied by replacing Step 4 with solving a set of equations to determine the multipliers λ and μ introduced due to the distortion and power constraints. In addition, the updated p(u,x) in Step 5 is based on the following form:*

$$\begin{array}{c} p_{k}\left(u,x\right)=\frac{e^{d\left[q_{k}\right]\left(u,x\right)-\lambda_{k}w_{k-1}\left(u,x\right)-\mu x^{2}}}{\sum e^{d\left[q_{k}\right]\left(u,x\right)-\lambda_{k}w_{k-1}\left(u,x\right)-\mu x^{2}}}. \end{array}$$

**Remark** **2.** *Note that the proposed algorithm does not require traversing the multiplier and can directly calculate the corresponding communication rate for a given distortion, which improves the defects of the classic AB algorithm, including the AB-type algorithm used to calculate the capacity–distortion trade-off of a monostatic ISAC system [6] (Theorem 4).*

### 3.2. Algorithm for Optimization Problem *(4)*

In this subsection, we apply the proposed framework to solve the optimization problem involving a log-loss distortion constraint. Following the framework discussed in the previous subsection, we replace the estimator p(s|u,z) in the optimization problem (4) with a free function f(s,u,z). Then, similar to the results above, we get that the optimal solution of the original optimization problem (4) is the same as that of the optimization problem as

maxp(u,x),f(u,s,z)maxq(·|·)∑p(u,x)p(y,s,z|x)lnq(x|u,y)q(u|z)p(u,x)s.t.−∑p(u,x)p(z,s|x)lnf(u,s,z)≤D,∑p(u,x)=1,∑sf(u,s,z)=1.

In addition, the optimal q(·|·) satisfies

(15) $$\begin{array}{c} q(x|u,y)=p(x|u,y)\;\mathrm{and}\;q(u|z)=p(u|z), \end{array}$$

the optimal f(u,s,z) satisfies

(16) $$\begin{array}{c} f(u,s,z)=p(s|u,z), \end{array}$$

and the optimal p(u,x) satisfies

(17) $$\begin{array}{c} p\left(u,x\right)=\frac{e^{d[q](u,x)+\lambda w(u,x)}}{\sum_{u,x}e^{d[q](u,x)+\lambda w(u,x)}}, \end{array}$$

where $d\left[q\right]\left(u,x\right)=\sum_{y,z}p\left(y,z|x\right)\ln q\left(x|u,y\right)q\left(u|z\right)$, $w\left(u,x\right)=\sum_{s,z}p\left(z,s|x\right)\ln f\left(s,u,z\right)$, and λ satisfies

(18) $$\begin{array}{c} G_{l}\left(\lambda \right):=D+\frac{\sum e^{d[q](u,x)+\lambda w(u,x)}w\left(u,x\right)}{\sum e^{d[q](u,x)+\lambda w(u,x)}}=0. \end{array}$$

Similar to the analysis in the previous section, we obtain that the equation $G_{l}\left(\lambda \right)=0,\lambda \geq 0$ has a unique solution when *D* is achievable. In addition, the minimum achievable distortion is $D_{\min}^{l}=\min_{p(x)}H\left(S|X,Z\right)$, and the maximum achievable distortion is $D_{\max}^{l}=H\left(S|Z\right)$, where the input distribution p(x) is selected to maximize the communication rate I(X;Y). Thus, we obtain the extended AB algorithm in Algorithm 2 to solve the optimization problem (4), the details of which are omitted for convenience.
**Algorithm 2** Extended AB algorithm for the optimization problem (4)**Input:** 
p(y|x), p(z|x,s), p(s).  1:Compute $D_{\min}^{l}$ and $D_{\max}^{l}$ and select $D\in [D_{\min}^{l},D_{\max}^{l}]$.  2:Initialize p(u,x),f(s,u,z).  3:**while** not converge **do**  4:   Update q(x|u,y) and q(u|z) based on (15).  5:   Solve λ based on the equation (18).  6:   Update p(u,x) based on (17).  7:   Update f(s,u,z) based on (16).  8:**end while****Output:** 
p(u,x).

### 3.3. Convergence Analysis

In this subsection, we primarily demonstrate the convergence of Algorithm 1. Let $p_{n-1}$ and $q_{n-1}$ be the values of the variables p(u,x) and $\left(q(x|u,y,s),q(u|z)\right)$ at the (n−1)-th iteration of the algorithm, respectively. We have the following result for the function values $\tilde{F}$ in (7) generated in the iterations.

**Theorem** **2.** *The function values $\tilde{F}$ generated in the iterations of Algorithm 1 are monotonically non-decreasing and bounded, which satisfy $\tilde{F}(p_{n-1},$$q_{n-1})\leq \tilde{F}\left(p_{n-1},q_{n}\right)\leq \tilde{F}\left(p_{n},q_{n}\right)$.*

**Proof of Theorem** **2.** For convenience, we omit the variables corresponding to the probability distribution when there is no ambiguity. Recalling the definition of $\tilde{F}\left(p,q\right)$ in (7) and the update expression of *q* in (9), we get

$$\begin{array}{cc} \; & \tilde{F}\left(p_{n-1},q_{n}\right)-\tilde{F}\left(p_{n-1},q_{n-1}\right)\\ = & \sum p_{n-1}p\left(y,z,s|x\right)\log\frac{p_{n-1}\left(x|u,y,s\right)p_{n-1}\left(u|z\right)}{q_{n-1}\left(x|u,y,s\right)q_{n-1}\left(u|z\right)}\\ = & \sum p_{n-1}\left(u,y,s\right)p_{n-1}\left(x|u,y,s\right)\log\frac{p_{n-1}\left(x|u,y,s\right)}{q_{n-1}\left(x|u,y,s\right)}\\ \; & +\sum p_{n-1}\left(z\right)p_{n-1}\left(u|z\right)\log\frac{p_{n-1}\left(u|z\right)}{q_{n-1}\left(u|z\right)}\\ = & \sum p_{n-1}\left(u,y,s\right)D\left(p_{n-1}\left(x|u,y,s\right)\left|\right|q_{n-1}\left(x|u,y,s\right)\right)\\ \; & +\sum p_{n-1}\left(z\right)D\left(p_{n-1}\left(u|z\right)\left|\right|q_{n-1}\left(u|z\right)\right)\\ \geq & \;0, \end{array}$$

where $p_{n-1}\left(x|u,y,s\right)$ refers to the conditional density function generated by $p_{n-1}$, i.e., $p_{n-1}\left(x|u,y,s\right)=p_{n-1}p\left(y,s|x\right)/\sum_{x}p_{n-1}p\left(y,s|x\right)$, and the others are similar.

According to the update expression of p(u,x) in (12), we obtain that

$$\begin{array}{cc} \; & \tilde{F}\left(p_{n},q_{n}\right)-\tilde{F}\left(p_{n-1},q_{n}\right)\\ = & \sum p_{n}\left(d\left[q_{n}\right]-\log p_{n}\right)-\sum p_{n-1}\left(d\left[q_{n}\right]-\log p_{n-1}\right)\\ = & \sum p_{n}\lambda_{n}w_{n-1}+\log\sum e^{d\left[q_{n}\right]-\lambda_{n}w_{n-1}}-\sum p_{n-1}\left(d\left[q_{n}\right]-\log p_{n-1}\right)\\ = & \sum p_{n}\lambda_{n}w_{n-1}+\sum p_{n-1}\log\sum e^{d\left[q_{n}\right]-\lambda_{n}w_{n-1}}-\sum p_{n-1}(d\left[q_{n}\right]-\lambda_{n}w_{n-1}+\lambda_{n}w_{n-1}\\ \; & \;\;-\log p_{n-1})\\ = & \sum p_{n}\lambda_{n}w_{n-1}-\sum p_{n-1}\log p_{n}-\sum p_{n-1}\lambda_{n}w_{n-1}+\sum p_{n-1}\log p_{n-1}\\ = & D(p_{n-1}\left|\right|p_{n})+\lambda_{n}\sum p_{n}w_{n-1}-\lambda_{n}\sum p_{n-1}w_{n-1}. \end{array}$$

Due to the fact that λ satisfies G(λ)=0, we have

$$\begin{array}{c} \sum p_{n}w_{n-1}=\sum p_{n}p\left(z,s|x\right){\left(s-c_{n-1}\right)}^{2}=\sum p_{n-1}p\left(z,s|x\right){\left(s-c_{n-2}\right)}^{2}=\sum p_{n-1}w_{n-2}=D. \end{array}$$

Therefore, we get

$$\begin{array}{cc} \; & \tilde{F}\left(p_{n},q_{n}\right)-\tilde{F}\left(p_{n-1},q_{n}\right)\\ = & D(p_{n-1}\left|\right|p_{n})+\lambda_{n}\sum p_{n}w_{n-1}-\lambda_{n}\sum p_{n-1}w_{n-1}\\ = & D(p_{n-1}\left|\right|p_{n})+\lambda_{n}\sum p_{n-1}\left(w_{n-2}-w_{n-1}\right)\\ = & D(p_{n-1}\left|\right|p_{n})+\lambda_{n}\sum p_{n-1}p\left(z,s|x\right){\left(s-c_{n-2}\right)}^{2}-\lambda_{n}\sum p_{n-1}p\left(z,s|x\right){\left(s-c_{n-1}\right)}^{2}\\ \geq & \;0, \end{array}$$

where the last inequality holds according to the fact that $c_{n-1}$ is the optimal estimator when the corresponding input distribution is $p_{n-1}$, i.e., its corresponding distortion is minimized. Furthermore, we have $\tilde{F}\left(p,q\right)\leq F\left(p\right)\leq I\left(X;Y|S\right)+I\left(X;Z\right)$, which completes the proof.    □

Unfortunately, due to the non-convex nature of the optimization problem, whether this algorithm can converge to the global optimal solution remains an open question.

## 4. Extension to Lossy Source Coding with Side Information at Decoder

**Figure 4:**
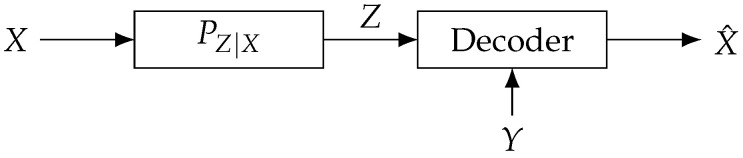
Source coding with side information at the decoder (see Figure 2 in [19]).

The optimization problem formulated for the bistatic ISAC system has a similar form to the lossy source coding problem with side information available at the decoder. Given this structural similarity, our proposed algorithm for the bistatic ISAC problem can be adapted to solve this related source coding problem. Consider the problem of source coding, where the decoder has access to side information about the source. As shown in Figure 4, *X* is a source, *Z* is a compressed version of source *X*, and the decoder produces an estimate $\hat{X}$ of the source *X* based on *Z* and the side information *Y* related to source *X*. The system aims to minimize the compression ratio *R* from *X* to *Z* while ensuring that the distortion between *X* and $\hat{X}$ remains less than a given value. Regarding this issue, the following conclusion holds.

**Lemma** **6** (Theorem 1, [19]). *The capacity–distortion function R(D) for the above system is the optimal solution to the following optimization problem:*

(19) $$\begin{array}{cc} \min_{P_{Z|X}} & \;I(X;Z|Y)\\ s.t. & \;E[d\left(X,\hat{X}\left(Y,Z\right)\right)]\leq D, \end{array}$$

*where the joint distribution of XYZ is given by $P_{XY}P_{Z|X}$.*

For the optimization problem (19), if SE is selected as the distortion function, the corresponding optimization problem is shown in (20) as

(20) $$\begin{array}{cc} \min_{P_{Z|X}} & \;I(X;Z|Y)\\ s.t. & \;E\left[{\left(X-\hat{X}\left(Y,Z\right)\right)}^{2}\right]\leq D. \end{array}$$

where

$$\begin{array}{cc} \; & \hat{X}\left(Y=y,Z=z\right)=E\left[X|Y=y,Z=z\right]\\ = & \sum_{x}P_{X|YZ}\left(x|y,z\right)x=\frac{\sum_{x}P_{XY}\left(x,y\right)P_{Z|X}\left(z|x\right)x}{\sum_{x}P_{XY}\left(x,y\right)P_{Z|X}\left(z|x\right)}. \end{array}$$

It is observed that the constraint is non-convex with respect to the optimization variable, distinguishing it from the classic source coding problem, which leads to the traditional AB algorithm used for solving rate-distortion problems being inapplicable. Given its structural similarity to the optimization problem (2), the proposed extended AB algorithm can be employed to solve it. The corresponding procedure is provided in Appendix A.

Furthermore, if log-loss is selected as the distortion function, the corresponding optimization problem is shown in (21) as

(21) $$\begin{array}{cc} \min_{P_{Z|X}} & \;I(X;Z|Y)\\ s.t. & \;H(X|Y,Z)\leq D. \end{array}$$

A variation of this problem is the optimization problem with mutual information constraints, which is shown as follows:

(22) $$\begin{array}{cc} \min_{P_{Z|X}} & \;I(X;Z|Y)\\ s.t. & \;I(X;Y,Z)\geq I, \end{array}$$

and the two optimization problems are equivalent when I=H(X)−D. The problem (22) resembles the information bottleneck problem and thus can be solved by leveraging related optimization algorithms [20,21,22].

## 5. Numerical Simulations

In this section, we evaluate the performance of the proposed algorithms for bistatic ISAC systems and the source coding problem with side information, respectively. In order to facilitate comparison with existing conclusions, in the simulations, we set the base of the logarithmic function to 2. To apply the extended AB algorithm to a continuous distribution, discretization is necessary. In the subsequent simulations, we fix the input power at B=10 dB and uniformly discretize the input *X* over the range [−10:q:10], where *q* is the step size. For a Gaussian distribution $N(\mu ,\sigma^{2})$, we truncate its range to the interval [μ−5σ,μ+5σ] and quantize it to [μ−5σ:q:μ+5σ]. Furthermore, the multipliers are solved for directly using the fsolve function from Optimization Toolbox in MATLAB 2023b.

### 5.1. State-Sum Gaussian Channel for ISAC Systems

Consider an additive Gaussian channel model with a power constraint for the bistatic ISAC system. The channel from the ISAC Tx to the ComRx is $Y=S+X+N_{1}$ and the channel from the ISAC Tx to the SenRx is $Z=Y+N_{2}$, where *S*, $N_{1}$, and $N_{2}$ are independently generated from $S∼N\left(0,\sigma_{s}^{2}\right),N_{1}∼N\left(0,\sigma_{1}^{2}\right)$, and $N_{2}∼N\left(0,\sigma_{2}^{2}\right)$. For this channel model with $\sigma_{s}^{2}=\sigma_{1}^{2}=\sigma_{2}^{2}=1$, we have the following quantization schemes: the input *X* is quantized as $X_{q}=\left[-10:q:10\right]$, the state *S* is quantized as $S_{q}=\left[-5:q:5\right]$, and both noise terms $N_{1}$ and $N_{2}$ are quantized as $N_{q}=\left[-5:q:5\right]$. Consequently, the output *Y* is quantized to $Y_{q}=S_{q}+X_{q}+N_{q}$, and the quantized output *Z* shares the same alphabet as $Y_{q}$. Furthermore, Algorithm 1 indicates that the computational complexity of the algorithm is of order O(|U||S||X||Y|), where |X| denotes the size of the alphabet of variable *X*. Under the given channel model and the quantization setup, this corresponds to an approximate complexity of $O(10^{4}q^{-4})$.

**Figure 5:**
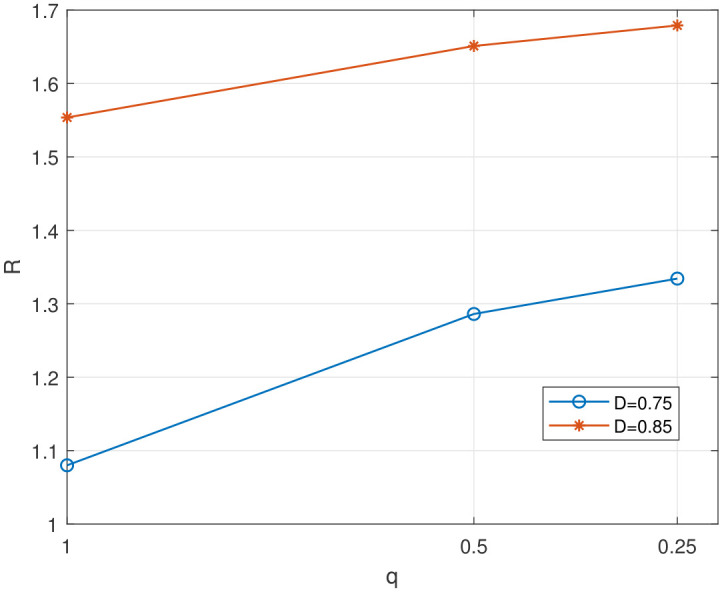
The impact of quantization step size on the value of rate.

In Figure 5, we evaluate the impact of the quantization step size *q* on the value of rate for a given distortion. It is observed that a smaller *q* (meaning a finer discretization grid) accordingly leads to a larger rate value. However, considering the computational complexity of the algorithm and hardware limitations, we set q=0.25 in the subsequent simulations.

**Figure 6:**
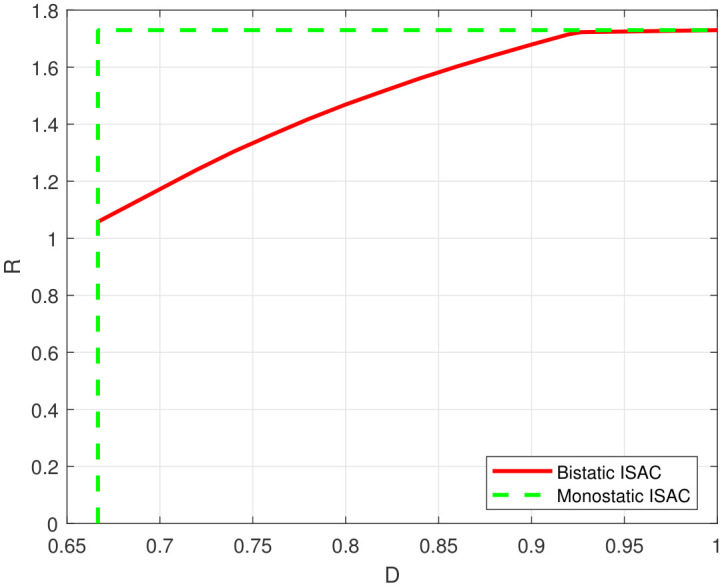
Rate-distortion function of different ISAC systems with SE distortion constraint.

By applying the proposed extended AB algorithm to the discretized model, we obtained the rate-distortion functions for a monostatic ISAC system and a bistatic ISAC system shown in Figure 6. In a monostatic ISAC system, the estimator is aware of *X*, resulting in the distortion being Var(S|X,Z)=2/3, which is independent of the distribution of *X*. On the other hand, the rate is I(X;Y|S), which reaches a maximum value $1/2\log_{2}11=1.7297$ when X∼N(0,10). As illustrated in Figure 6, the capacity–distortion function for the monostatic ISAC system is located to the upper left of the capacity–distortion function of the bistatic system, indicating that the system suffers from a performance loss when the estimator lacks knowledge of the sent information.

For the case where the distortion metric is log-loss, we replace the channel from the ISAC Tx to the ComRx in the additive Gaussian channel model above with $Y=X+N_{1}$ to be consistent with the model in [12]. For this additive Gaussian channel model, the authors of [12] presented a closed-form solution under the condition that $\sigma_{1}^{2}\leq \sigma_{2}^{2}$. Additionally, they provided a lower bound for the optimal solution when $\sigma_{2}^{2}\leq \sigma_{1}^{2}\leq \sigma_{2}^{2}+\sigma_{s}^{2}$.

**Figure 7:**
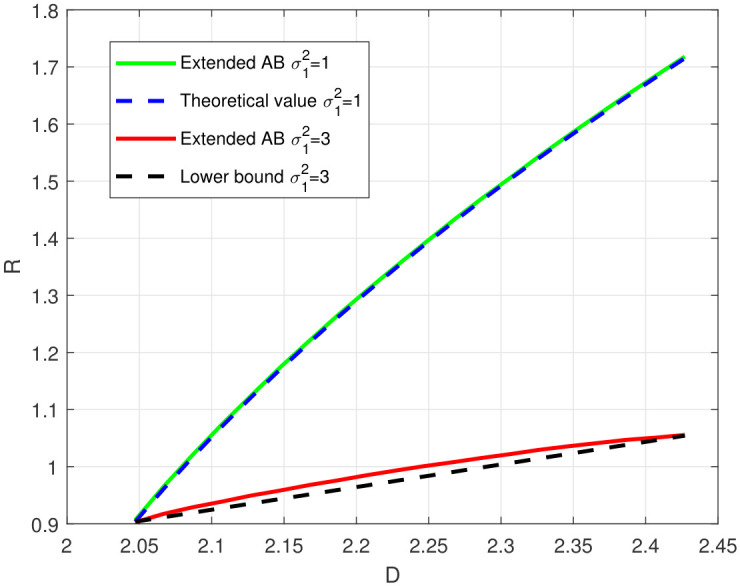
Rate-distortion function of the bistatic ISAC system with log-loss distortion constraint.

Figure 7 depicts the rate-distortion functions of the bistatic ISAC system with the log-loss distortion constraint under different parameters. Specifically, the channel parameters satisfy $\sigma_{s}^{2}=2$, $\sigma_{2}^{2}=2$, and B=10, and $\sigma_{1}^{2}=1<\sigma_{2}^{2}$ for the first case and $\sigma_{2}^{2}<\sigma_{1}^{2}=3<\sigma_{2}^{2}+\sigma_{s}^{2}$ for the second case. It is observed that the rate-distortion function curve obtained by the algorithm aligns well with the theoretical value for the first case, and the obtained rate-distortion function curve outperforms the theoretical lower bound [12] for the second case. In summary, the effectiveness of the proposed algorithm is verified.

### 5.2. State-Product Gaussian Channel for ISAC Systems

Consider a real Gaussian channel with Rayleigh fading, which incorporates a multiplicative Gaussian state. Specifically, the channel from the ISAC Tx to the ComRx is $Y=SX+N_{1}$, and the channel from the ISAC Tx to the SenRx is $Z=Y+N_{2}$, where *S*, $N_{1}$, and $N_{2}$ are independently generated from $S∼N\left(0,\sigma_{s}^{2}\right),N_{1}∼N\left(0,\sigma_{1}^{2}\right)$, and $N_{2}∼N\left(0,\sigma_{2}^{2}\right)$. For this channel model with $\sigma_{s}^{2}=\sigma_{1}^{2}=\sigma_{2}^{2}=1$, we adopt a quantization scheme similar to that used for the state-sum Gaussian channel, except that both noise terms $N_{1}$ and $N_{2}$ are quantized to $N_{q}=\left[-5:q^{2}:5\right]$ due to the multiplicative nature of the channel. The resulting algorithmic complexity is approximately $O(10^{5}q^{-5})$.

**Figure 8:**
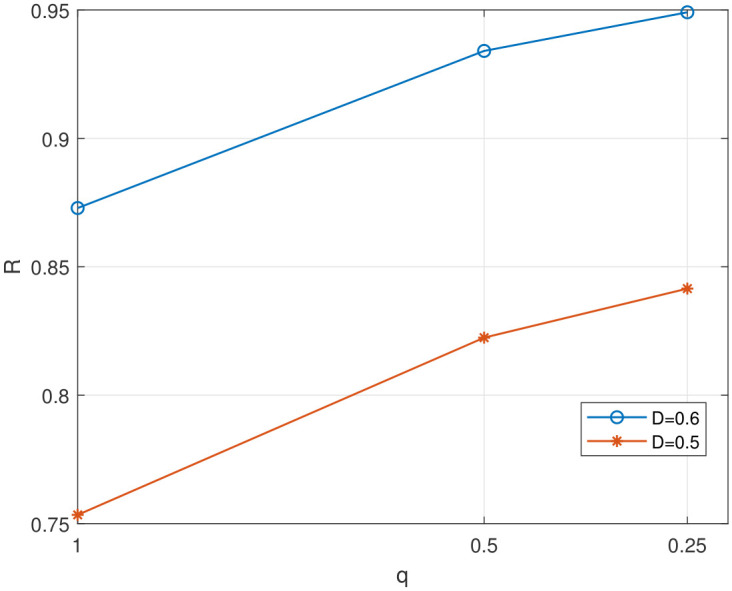
The impact of quantization step size on the value of rate.

In Figure 8, we evaluate the impact of quantization step size *q* on the value of rate for a given distortion. Considering the computational complexity of the algorithm and hardware limitations, we set q=0.25 in the subsequent simulations.

**Figure 9:**
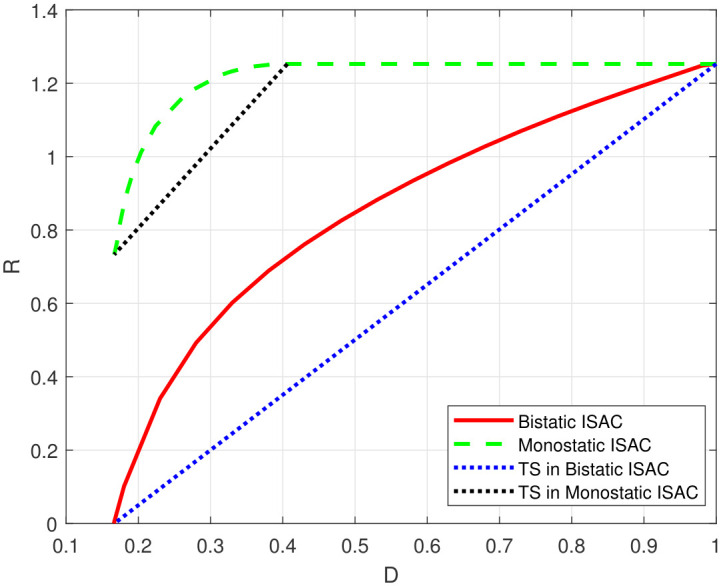
Capacity–distortion functions for state-product Gaussian channel with SE distortion constraint.

In Figure 9, we compare the capacity–distortion functions of the state-product Gaussian channel model for a monostatic ISAC system, a bistatic ISAC system, and the time-sharing (TS) schemes. The TS scheme refers to independent communication and sensing in a time-sharing manner. For the monostatic ISAC system, we plotted its capacity distortion function by exploiting the AB type algorithm proposed in [6]. For the bistatic ISAC system, the SenRx estimates the state *S* from the received signal under the condition of unknown sent signal *X*. We plotted its capacity distortion function according to the proposed extended AB algorithm. In addition, for both monostatic and bistatic ISAC systems, the input distribution of the endpoints in the TS scheme is identical. In this scheme, the lower-left endpoint is achieved via 2-ary pulse amplitude modulation (PAM), while the upper-right endpoint is realized with a Gaussian input X∼N(0,10). As illustrated in Figure 9, the capacity–distortion function for the monostatic ISAC system is located to the upper left of the capacity–distortion function of the bistatic system. The performance degradation in the bistatic configurations stems from the spatial separation of the transmitter and receiver. This separation deprives the estimator of the direct knowledge of the transmitted signal, which is inherently available in a monostatic system. The simulations were initialized with a random matrix and stopped when the 2-norm difference between successive input distribution matrices was less than $10^{-5}$. Furthermore, it was observed that the results from multiple trials were nearly consistent, with rate value discrepancies not exceeding $10^{-3}$, despite a completely random assignment of initial values. The consistency confirms the weak dependence of the simulation results on the chosen initial conditions.

### 5.3. Source Coding Problem with Side Information

In this subsection, we verify the effectiveness of our algorithm for the source coding problem in Section 4. Specifically, we consider the cases of Gaussian sources, uniform sources, and mixed Gaussian sources. For Gaussian sources, let $X∼N(0,\sigma_{x}^{2})$, and Y=X+N, where *X* and *N* are independent, with $N∼N(0,\sigma^{2})$. The authors of [19] provided a closed-form expression for the optimal solution as follows:

(23) $$\begin{array}{c} R\left(D\right)=\left\{\begin{array}{cc} \frac{1}{2}\log\frac{\sigma^{2}\sigma_{x}^{2}}{(\sigma^{2}+\sigma_{x}^{2})D}, & 0\leq D<\frac{\sigma^{2}\sigma_{x}^{2}}{\sigma^{2}+\sigma_{x}^{2}},\\ 0, & D\geq \frac{\sigma^{2}\sigma_{x}^{2}}{\sigma^{2}+\sigma_{x}^{2}}. \end{array}\right. \end{array}$$

**Figure 10:**
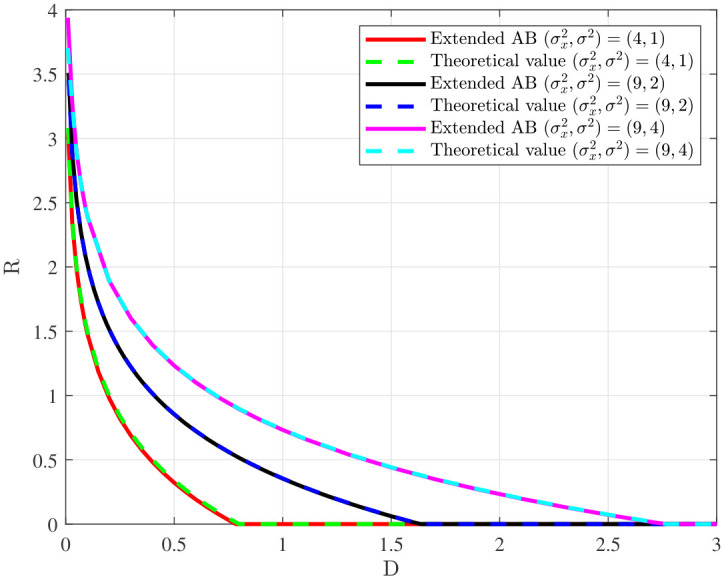
Rate-distortion function of Gaussian source.

In Figure 10, we compare the proposed extended AB algorithm with the theoretical value in (23) for a Gaussian source. It is observed that the rate-distortion curve obtained by the algorithm is consistent with the theoretical value, which verifies the effectiveness of the algorithm.

In the following, we consider the cases where the source is uniformly distributed and mixed Gaussian distributed. Assume Y=X+N, where *X* and *N* are independent, with N∼N(0,1) for convenience.

**Figure 11:**
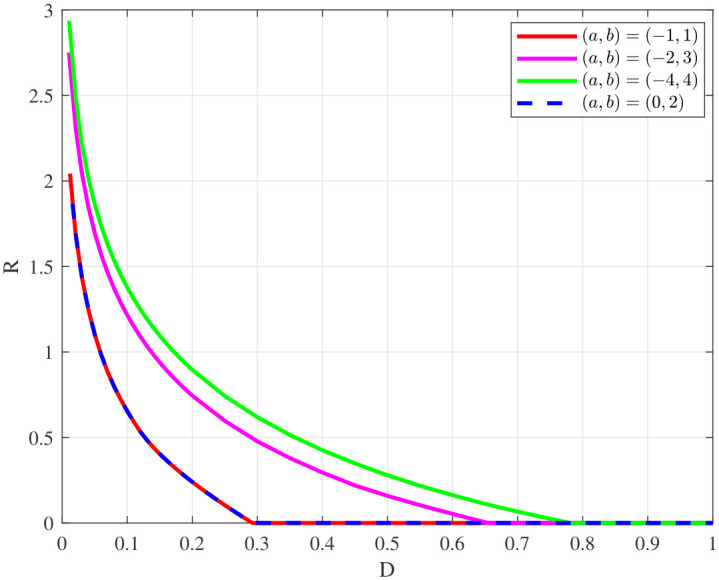
Rate-distortion function of uniform source.

Figure 11 illustrates the rate-distortion function of the uniform source where the sources *X* satisfy X∼Unif(a,b) where a,b∈R. It is observed that under this model setting, the rate-distortion function of the uniform source only remains related to its interval length, i.e., |b−a|. Moreover, the longer the interval length, the more to the right the corresponding rate-distortion curve is.

**Figure 12:**
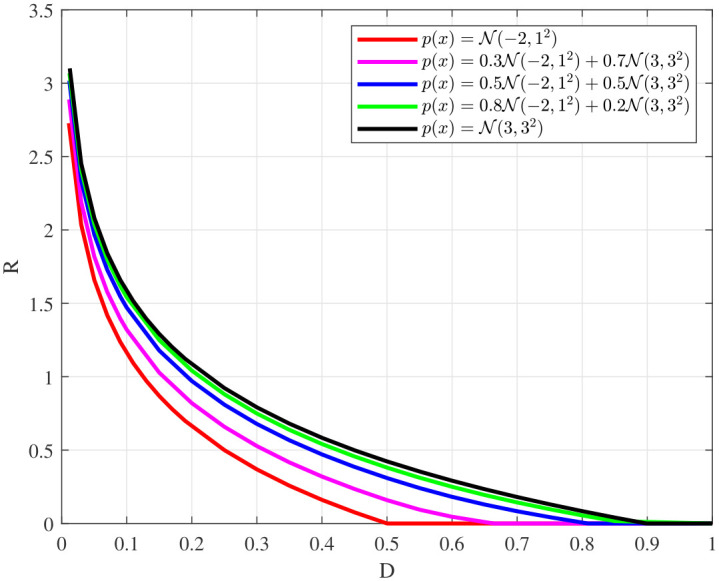
Rate-distortion function of mixed Gaussian source.

Figure 12 depicts the rate-distortion function of the mixed Gaussian sources, which are composed of Gaussian distribution $N(-2,1^{2})$ and Gaussian distribution $N(3,3^{2})$ with different weights. It is observed that from left to right in Figure 12, the weight of Gaussian distribution $N(3,3^{2})$ in the mixed Gaussian distribution increases continuously, and the corresponding rate-distortion curve gradually approaches that of Gaussian distribution $N(3,3^{2})$, which is consistent with our expectations.

## 6. Conclusions

In this paper, we proposed an extended AB framework to solve capacity–distortion functions for bistatic ISAC systems. Specifically, for the corresponding non-convex optimization problems with SE and log-loss distortion constraints, we introduced auxiliary variables to transform the original non-convex constraints into linear forms, while ensuring that the reformulated linearly constrained optimization problems maintained the same optimal solution as the original problems. Building on the AB algorithm framework, we then developed extended AB algorithms for the reformulated optimization problems that retain closed-form variable updates while overcoming the limitations of handling non-convex constraints. Furthermore, we extended the proposed algorithm to solve lossy source coding problems with side information. Numerical simulations are presented, which validate the effectiveness of the proposed algorithms and provide a visual comparison of the performance in bistatic versus monostatic ISAC systems.

## Data Availability

The original contributions presented in the study are included in the article; further inquiries can be directed to the first author.

## Appendix A. Algorithm for Optimization Problem in Source Coding with Side Information

In the following, we apply the proposed framework to solve the optimization problem (20). Specifically, following the idea of the AB algorithm, we write the objective function of the original optimization problem (20) as

$$\begin{array}{c} I\left(X;Z|Y\right)=\sum p\left(x,y\right)p\left(z|x\right)\log\frac{p(z|x)}{p(z|y)} \end{array}$$

and define

(A1) $$\begin{array}{c} \tilde{F}\left(p(z|x),q(z|y)\right)=\sum p\left(x,y\right)p\left(z|x\right)\log\frac{p(z|x)}{q(z|y)}, \end{array}$$

which is a convex function of the optimization variable p(z|x) for fixed q(z|y). Furthermore, we introduce a free variable $c_{s}\left(y,z\right)$ to replace the estimator $\hat{X}\left(y,z\right)$. Then, similar to the analysis in Section 3, we get that the optimal solution of the original optimization problem is the same as that of the optimization problem

maxp(z|x),cs(y,z)maxq(z|y)∑p(x,y)p(z|x)logp(z|x)q(z|y)s.t.∑p(x,y)p(z|x)(x−cs(y,z))2≤D,∑p(u,x)=1.

In addition, the optimal q(z|y) satisfies

(A2) $$\begin{array}{c} q\left(z|y\right)=\sum_{x}p\left(z|x\right)p\left(x|y\right), \end{array}$$

the optimal $c_{s}\left(y,z\right)$ satisfies

(A3) $$\begin{array}{c} c_{s}\left(y,z\right)=\frac{\sum_{x}p\left(z|x\right)p\left(x,y\right)x}{\sum_{x}p\left(z|x\right)p\left(x,y\right)}, \end{array}$$

and the optimal p(z|x) satisfies

(A4) $$\begin{array}{c} p\left(z|x\right)=\frac{e^{d[q](x,z)-\lambda w(x,z)}}{\sum_{z}e^{d[q](x,z)-\lambda w(x,z)}}, \end{array}$$

where $d\left[q\right]\left(x,z\right)=\sum_{y}p\left(y|x\right)\log q\left(z|y\right)$, $w\left(x,z\right)=\sum_{y}p\left(y|x\right){\left(x-c_{s}\left(y,z\right)\right)}^{2}$, and λ satisfies

(A5) $$\begin{array}{c} G_{s}\left(\lambda \right):=D-\sum_{x}p\left(x\right)\frac{\sum_{z}e^{d[q](x,z)-\lambda w(x,z)}w\left(x,z\right)}{\sum_{z}e^{d[q](x,z)-\lambda w(x,z)}}=0. \end{array}$$

Thus, an extended AB algorithm is developed to solve the optimization problem for source coding with side information, as detailed in Algorithm A1.

**Algorithm A1** Extended AB algorithm for the optimization problem (20)
Input: p(x,y), p(z|x), $c_{s}\left(y,z\right)$, and *D* 1: **while** not converge **do** 2: Update q(z|y) based on (A2). 3: Solve λ based on the equation (A5). 4: Update p(z|x) based on (A4). 5: Update $c_{s}\left(y,z\right)$ based on (A3). 6: **end while** Output: p(z|x).
